# Effects of GV14 Acupuncture on Cerebral Blood Flow Velocity in the Basilar and Middle Cerebral Arteries and CO_2_ Reactivity during Hypercapnia in Normal Individuals

**DOI:** 10.1155/2021/9319413

**Published:** 2021-09-09

**Authors:** Hyun Ku Lee, Sang-Kwan Moon, Chul Jin, Seung-Yeon Cho, Seong-Uk Park, Woo-Sang Jung, Jung-Mi Park, Chang-Nam Ko, Ki-Ho Cho, Seungwon Kwon

**Affiliations:** ^1^Department of Korean Medicine Cardiology and Neurology, Graduate School, Kyung Hee University, Seoul, Republic of Korea; ^2^Department of Cardiology and Neurology, College of Korean Medicine, Kyung Hee University, Seoul, Republic of Korea

## Abstract

The Governing Vessel 14 (GV14) (Dazhui) is one of the acupuncture points referred to as “seven acupoints for stroke.” Nevertheless, there is a scarcity of research on the effects of acupuncture treatment at GV14. This study investigated the effects of acupuncture at GV14 on cerebral blood flow (CBF), especially that in the basilar artery (BA) and the middle cerebral arteries (MCA). Sixteen healthy men aged 20 to 29 years were enrolled in this study. CBF velocity and cerebrovascular reactivity (CVR) were measured using transcranial Doppler sonography (TCD). The following were assessed: closed circuit rebreathing- (CCR-) induced carbon dioxide (CO_2_) reactivity, modified blood flow velocity at 40 mmHg (CV40) on BA and MCAs, blood pressure (BP), and heart rate (HR). Observed results were obtained after comparison with the baseline evaluation. Statistically significant elevations in CO_2_ reactivity were recorded in the BA (3.28 to 4.70, *p* < 0.001) and MCAs (right: 3.81 to 5.25, *p*=0.001; left: 3.84 to 5.12, *p*=0.005) after acupuncture at GV14. The CV40 increased statistically significantly only in the BA (45.49 to 50.41, *p*=0.003). No change was observed in BP (106.83 to 107.08 (mmHg), *p*=0.335) and HR (77 to 75 (bpm), *p*=0.431). Acupuncture at GV14 improved CBF velocity. These results could be explained by the regulation of endothelium-dependent vessel dilation effected by acupuncture. This trial is registered with Korean Clinical Trial Registry (http://cris.nih.go.kr; registration number: KCT0004787).

## 1. Introduction

Acupuncture has been used for the treatment of a variety of diseases, including stroke, for thousands of years. Several recent trials have shown that acupuncture has the potential to become valuable for the treatment [1–3], rehabilitation [4], and prevention of stroke [5, 6].

Several acupuncture mechanisms have been discovered to improve stroke, one of which involves increase in the cerebral blood flow (CBF) as demonstrated by positron emission tomography (PET), single-photon emission computed tomography (SPECT), and transcranial Doppler (TCD) [7–12].

TCD is highly advantageous because of the ease of the accessibility, i.e., the test is less time-consuming and portable, cost-effective, low risk, and noninvasive [13, 14]. Moreover, it enables real-time flow monitoring, continuous measurement, and assessment of cerebral autoregulation in the laboratory or bedside using a dynamic approach [15]. TCD has been used to support the evidence of the prognostication and initiation of preventive strategies in diseases caused by impairment of the cerebrovascular autoregulation, vasoreactivity, and cerebral hyperperfusion such as sickle cell disease [16], subarachnoid hemorrhage [17], stroke [18], dementia [19], vascular depression [20], and Fabry disease [21]. Several previous studies using TCD have found CBF changes after acupuncture stimulation at specific acupuncture points, such as GV20 [22], GB20 [23], ST36 [24], GB34 [25], LI11 [7], and GB39 [8].

GV14 is the 14th acupoint on the Governing Vessel meridian. This meridian lies along the posterior median line and leads yang meridians throughout the body. Traditionally, GV14 has been included in acupuncture points known as the “seven acupoints for stroke,” which are used to treat the symptoms of stroke. Some clinical trials evaluated the effect of GV14 acupuncture on CBF. Litscher et al. [26] and Wang et al. [27] used violet laser acupuncture at GV14 in healthy individuals and found the CBF velocity increased in the BA along with improvements in the microcirculation of blood flow. Kim et al. [28] attempted electroacupuncture stimulation on GV20 and GV14 in ischemic mice and found that it increased blood perfusion to the cerebral cortex. Jeong [29] used pharmacopuncture therapy using Carthami Flos at GV14 in healthy and ischemia rats and found that the CBF was increased in healthy rats, while it enhanced the stability of CBF in the ischemic state. Zhang et al. [30] attempted acupuncture at GV14 in the cerebral ischemia rabbit model and used functional magnetic resonance imaging (fMRI) to examine the sites of brain activation. They found increased blood flow to the site of brain ischemia and peripheral tissues.

However, despite these various clinical uses, there was no study that has focused solely on the role of GV14 acupuncture for the CBF in humans. Therefore, this study aimed to evaluate the effect of acupuncture at GV14 on CO_2_ reactivity in the cerebral arteries using TCD. We hypothesized that acupuncture at GV14 could improve CBF in BA and both MCAs.

## 2. Methods

The present study was designed as a single-center, before-and-after study. This study was approved by the Institutional Review Board at the Hospital of Korean Medicine, Kyung Hee Medical Center (KOMCIRB 2019-11-002-001), and registered with Clinical Research Information Service, a service of the Korea Centers for Disease Control and Prevention (KCT0004787).

### 2.1. Participants

Participants were recruited via in-hospital advertisement. Sixteen healthy male volunteers aged 20 to 29 years were enrolled in this study. All participants were informed of the procedures and signed a written informed consent form before enrollment. None of the participants had a history of cerebrovascular disease, cardiovascular disease, diabetes mellitus, hypertension, endocrinologic disease, or psychiatric problems. Moreover, the participants did not have any diseases during the study period. They were prohibited from consuming caffeine, alcohol, and drugs for 24 hours before the study. Participant recruitment was conducted between November 2019 and October 2020.

### 2.2. Sample Size Calculation

The sample size was calculated to enhance the reliability of the results. Sample size calculation was performed using G∗power, version 3.1.9.4. No previous study has used GV14 acupuncture in humans. Thus, the sample size was calculated by referring to the results of a previous study using GB20 [23]. The main outcome was the change in the CO_2_ reactivity of the BA before and after GV14 acupuncture. According to the previous study [23], the CO_2_ reactivity of the BA was 1.8 ± 0.75%/minute in the before acupuncture group and 2.5 ± 0.89%/minute in the after acupuncture group. Taking into consideration a two-sided significance level of 5% (*α*) and a test power of 80% (*β*), the required sample size was 14. Moreover, the required sample size was 16, considering a 10% dropout rate.

### 2.3. Acupuncture Treatment at GV14

All procedures of acupuncture treatment were performed by an experienced Korean medical doctor with more than 3 years of experience. The acupuncture point, GV14, is located on the posterior region of the neck, in the depression inferior to the spinous process of the 7th cervical vertebra (C7), along the posterior median line. The location of the GV14 was determined according to the WHO standard acupuncture point locations [31]. A stainless steel acupuncture needle (diameter 0.25 mm, length 40 mm; DongBang Acupuncture, Seoul, Korea) was used for GV14 acupuncture. The GV14 was stimulated manually with a twisting needle until the participant felt the De-Qi sensation (aching, soreness, pressure, fullness, heaviness, numbness, tingling, warmth, coolness, and dullness sensation) [32, 33]. The needle was inserted into the skin approximately 10 mm deep and the needle was removed after 20 minutes. All procedures were conducted in accordance with the Revised Standards for Reporting Interventions in Clinical Trials of Acupuncture (STRICTA [34]; Table 1) guidelines.

Each participant received acupuncture treatment twice, once at each visit.

### 2.4. Measurements

The research protocol was based on previous studies that used TCD to identify the relationship between blood flow in the cerebral arteries and acupuncture points [7, 8, 22–25, 35]. For each process, CBF velocity and the CO_2_ reactivity of the BA and both MCAs were measured using a Multi-Dop X4 system TCD device (Compumedics DWL, Singen, Germany).

Variables that can affect CBF, i.e., blood pressure, heart rate, and end-tidal carbon dioxide (*P*_ETCO2_), were measured using various modules on the Cardiocap S/5 monitor (Datex-Ohmeda, Helsinki, Finland). The mean blood pressure was determined. The heart rate was continuously recorded via an oximetry device attached to the participant's finger. Further, P_ETCO2_ was continuously obtained via a Cardiocap S/5 monitor-connected nasal prong placed in the participant's nostril, and each participant was instructed to breathe only through the nose during the procedure. A snapshot function in the Cardiocap S/5 monitor program was used to obtain the mean heart rate and P_ETCO2_ at specific time points during the procedure. These variables were monitored and recorded on a computer that was connected to the Cardiocap S/5 monitor program.

Each participant visited the study center twice with the interval of 1 week and received acupuncture treatment at each visit.

#### 2.4.1. Visit 1

**Figure 1:**
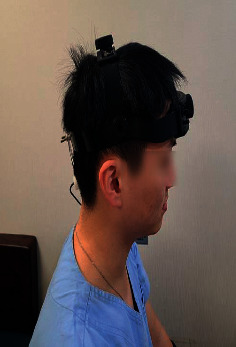
Suboccipital probe holder.

**Figure 2:**
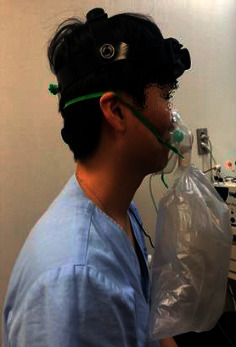
Suboccipital probe holder during closed circuit rebreathing.

**Figure 3:**
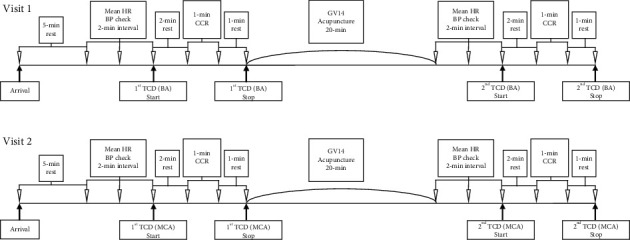
Timeline of study procedure. HR, heart rate; BP, blood pressure; TCD, transcranial Doppler; CCR, closed circuit rebreathing.

At the first visit, each participant was asked to sit in a comfortable position. The Cardiocap S/5 monitor and probe-holding device were positioned (refer to Figure 1) to ascertain the CBF velocities of BA through the suboccipital window using a 2 MHz pulsed-Doppler probe. The strongest wave pattern was captured at depths ranging from 75 to 110 mm for BA. All measurements were initiated after making the participant rest for 5 minutes. Each participant was asked to sit in a comfortable position. The Cardiocap S/5 monitor and probe-holding device was positioned (refer to Figure 1.) to ascertain the CBF velocities through the suboccipital window using a 2 MHz pulsed-Doppler probe. The blood pressure was checked 3 times every 2 minutes. After starting the first measurement of CBF velocity, the participant was allowed to take rest for 2 minutes and CCR was performed over 1 minute (see Figure 2). The CCR method entailed that participants inhaled their own exhaled air again using a 5-liter reservoir bag. After performing CCR, the participants were given a 1-minute break and the first TCD measurement was stopped. Then, acupuncture was performed at GV14 for 20 minutes. Subsequently, the same procedure was repeated for the postacupuncture measurements (see Figure 3 for the entire process). For TCD measurement, the sample and gain values were corrected and saved if the CBF wave patterns remained constant. The mean blood flow velocity was calculated continuously as the time-averaged maximum velocity over the cardiac cycle, as computed from the envelope of maximum frequencies. The mean blood flow velocities were obtained at rest under stable normocapnic conditions and near the end of the CCR period under hypercapnic conditions. All TCD spectra were recorded for subsequent review.

#### 2.4.2. Visit 2

**Figure 4:**
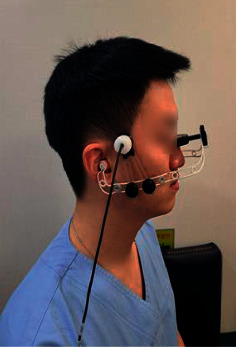
Bilateral probe holder.

**Figure 5:**
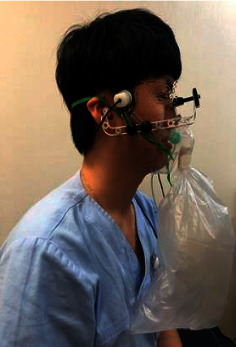
Bilateral probe holder during closed circuit rebreathing.

On the second visit, bilateral probe holder (LAM-Rack; Compumedics DWL) was attached at both temporal windows for each participant (see Figures 4 and 5) and CBF velocities of both MCAs were collected. The strongest wave pattern was captured at depths ranging from 40 to 60 mm for MCAs. For the measurement of all variables, the same processes as used at the first visit were repeated.

### 2.5. Calculations

CO_2_ reactivity refers to the percent change in mean blood flow velocity per millimeter of mercury change in P_ETCO2_, as calculated by the following formula [35]:

(1) $$\begin{array}{c} {\text{CO}}_{2}\text{ reactivity}=100\times \frac{\left[\left(V_{\text{hypercapnia}}-V_{\text{rest}}\right)/V_{\text{rest}}\right]}{\Delta P_{{\text{ETCO}}_{2}}}, \end{array}$$

where *V*_rest_ is the blood flow velocity at rest, obtained during the most stable period under stable normocapnic conditions; *V*_hypercapnia_ is the blood flow velocity in the latter half of the 1-minute CCR period; and Δ*P*_ETCO2_ is the change in *P*_ETCO2_ between baseline and maximal CCR.

CBF velocity is dependent on the arterial CO_2_ tension, and the corrected blood flow velocity was calculated at 40 mmHg of CO_2_ tension (CV40, cm/s) using the following formula [36]:

(2) $$\begin{array}{c} \text{CV}40\left(\text{corrected velocity at }P_{{\text{ETCO}}_{2}}\;40\text{ mmHg}\right)=V_{1}\cdot e^{b}{\;}^{\left(P{\text{CO}}_{2}\;40\text{mmHg}-P_{1}{\text{CO}}_{2}\right)}, \end{array}$$

where *b* represents CO_2_ reactivity; *V*_1_ represents velocity at P_1_CO_2_; and *P*_ETCO2_ represents the end-tidal CO_2_ partial pressure.

### 2.6. Statistical Analysis

Statistical analysis was performed using the Statistical Package for the Social Sciences version 25.0 for Windows (SPSS, Chicago, Illinois, United States). The data were summarized as the median (range). Statistical comparisons between the study parameters before and after GV14 acupuncture treatment were made using the Wilcoxon signed-rank test. *p* values under 0.05 were considered statistically significant. The data were summarized as the median (range).

## 3. Result

### 3.1. Changes of the CO_2_ Reactivity of the BA and Both MCAs after GV14 Acupuncture

**Figure 6:**
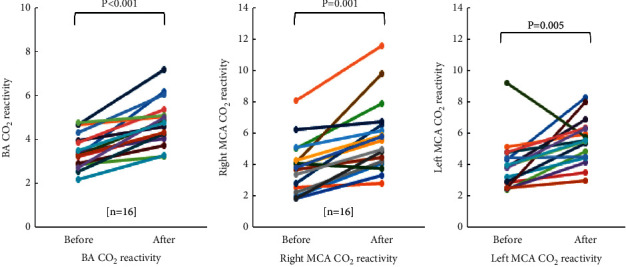
Changes in CO_2_ reactivity of BA and MCAs after the GV14 acupuncture. CO_2_ reactivity is significantly increased in BA and both MCAs after the GV14 acupuncture. BA, basilar artery; MCA, middle cerebral artery.

There was significant increase in the CO_2_ reactivity of the BA and both MCAs after GV14 acupuncture treatment compared with baseline (Table 2; Figure 6).

### 3.2. Changes of the CV40 of the BA and Both MCAs after GV14 Acupuncture

**Figure 7:**
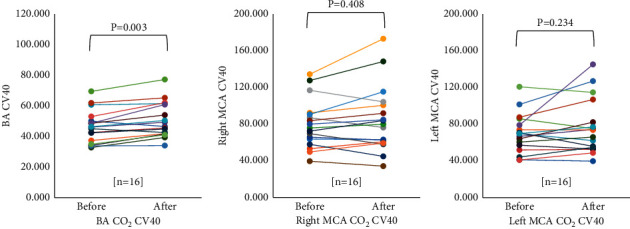
Changes in CV40 velocity (cm/s) of BA and MCAs after the GV14 acupuncture. CV40 velocity is significantly increased only in BA after the GV14 acupuncture (*p*=0.003). BA, basilar artery; MCA, middle cerebral artery.

There was a significant increase in the CV40 of the BA (*p*=0.003) after GV14 acupuncture. However, there were no significant changes in the CV40 between before and after GV14 acupuncture in both the MCAs (Table 3; Figure 7).

### 3.3. Changes of the Mean Blood Pressure and Heart Rate after GV14 Acupuncture

There was no significant difference in the mean blood pressure and heart rate before and after GV14 acupuncture (Table 4).

## 4. Discussion

The purpose of the present study was to determine whether GV14 acupuncture treatment would cause responses to CBF velocity and CO_2_ reactivity of the BA and MCA during hypercapnia in normal subjects. The result showed that GV14 acupuncture treatment significantly increased the CO_2_ reactivity of the BA from 3.28 to 4.70 (*p* < 0.001), Rt. MCA from 3.81 to 5.25 (*p*=0.001), and Lt. MCA from 3.84 to 5.12 (*p*=0.005). In addition, CV40 of the BA also increased significantly after GV14 acupuncture treatment from 45.49 to 50.41 (*p*=0.003), while those of both MCAs remarkably increased but not reaching statistical significance. These results indicate that GV14 acupuncture is strongly associated with improvement of cerebral blood flow in both posterior and anterior circulations.

Cerebral vasomotor reactivity (CVR) is the compensatory potential of the vessels regulating blood flow to the brain and is represented as the percentage change in response to an arteriolar-dilating stimulus such as carbon dioxide (CO_2_) or acetazolamide [37, 38]. In this study, the CCR-induced hypercapnia method was applied to measure CVR, namely, CO_2_ reactivity. For CCR, participants inhaled their own exhaled air again to increase the partial pressure of CO_2_ in the inhaled air. By repeatedly breathing in a 5-liter reservoir bag, the end-expiratory CO_2_ concentration is gradually increased. Subsequently, the CBF velocity gradually increased and was stabilized after approximately 60 seconds [39] rebreathing. CBF change by and adjustment of the vessel diameter can be divided into endothelium-dependent and -nondependent. In previous studies, CBF and CVR were reported to be associated with endothelium dependence [40, 41]. Acupuncture is considered to have a beneficial effect on blood flow by treating endothelial dysfunction [42, 43]. A previous study reported that acupuncture enhanced endothelial function and vascular reactivity by regulating vasoconstrictors and vasodilators [43]. In addition, acupuncture treatments on certain acupuncture points were reported to have effects on the specific brain regions and cerebral arteries. When electroacupuncture was applied to ST36–ST41, GB34–GB39, and LI4–LI11, respectively, there was a distinct difference between the increased and decreased areas of CBF on SPECT and statistical parametric mapping (SPM) [44–46]. In studies using TCD, when different acupoints were stimulated, the velocities of different cerebral blood vessels were changed. It shows specific acupuncture produces specific reproducible quantifiable effects on blood flow velocity in brain arteries [47–49]. The defined acupoints are considered important because the classical theory of traditional oriental medicine claims that targeting an acupoint results in clinical effect. Further, stimulation of different acupuncture points has different effects [50–53].

There have been several previous studies, which measure the effect of acupuncture on cerebral blood flow using TCD. A study of acupuncture at GV20 [22] reported that CVR and CV40 of both MCAs and anterior cerebral arteries (ACAs) were significantly increased. A study of acupuncture at GB20 [23] reported that the CVR of the BA was significantly increased. A study of acupuncture at ST36 [24] reported that CVR of both the MCA and BA was significantly increased. A study of acupuncture at GB34 [25] reported that CVR of the ipsilateral MCA was significantly increased. A study of acupuncture at LI11 [7] reported that CVR of the contralateral MCA was significantly increased. A study of acupuncture at GB39 [8] reported that CVR of both the ACA and contralateral MCA and CV40 of the contralateral ACA was significantly increased. In these studies [7, 8, 22–25], the hyperventilation-induced hypocapnia method was applied to measure CO_2_ reactivity. However, when hyperventilation is performed during TCD monitoring, even though it is a suitable method, the probe attached on the holder can be shaken, which might make it difficult to measure the blood flow. In addition, some participants appealed that rapid hyperventilation was not easy to do. Therefore, in this study, the CCR was used to try to improve these problems and the results indicate that it is easier and produce more stable measure values than hyperventilation method.

Previous studies showed that CBF velocity of the BA was increased and microcirculation of blood flow was improved after violet laser acupuncture on GV14 in normal individuals [26, 27]. Electroacupuncture stimulation on GV20 and GV14 increased blood flow perfusion in the cerebral cortex [28]. Pharmacopuncture therapy using Carthami Flos at GV14 could increase CBF in the normal state and improve the stability of CBF in ischemic state [29]. Acupuncture stimulation at GV14 in rabbits with cerebral ischemia could increase blood flow to the brain ischemia site and peripheral tissues on fMRI [30]. However, there have been no studies regarding the effect of GV14 single acupuncture on cerebral blood flow in human participants. In this study, GV14 single acupuncture was conducted in humans. Therefore, the CVR of BA and both MCAs and CV40 of BA significantly increased.

A number of studies showed that impairment of brain perfusion and CBF is one of the mechanisms by which vascular disease may contribute to neurodegeneration such as dementia and vascular depression [20, 21, 54, 55]. Recently, many studies have been conducted to improve these impairments using complementary and alternative treatments [56]. Through this study, it is thought that GV14 acupuncture can be used for various cerebral blood flow disorders other than just stroke.

The limitation of this study is that there was an absence of a control group, which may have caused placebo bias. All participants were male. Females were excluded because the menstrual cycle could affect CBF [57]. Furthermore, calculating the sample size increased the reliability of the study. In addition, it is meaningful to objectively prove the single effect of acupuncture on GV14 in humans.

## 5. Conclusions

This study demonstrated that GV14 acupuncture treatment increased CO_2_ reactivity in the BA and both MCAs and increased corrected velocity at P_ETCO2_ 40 mmHg in the BA during hypercapnia in healthy participants.

These results may clinically support the use of GV14 to treat disorders of BA and MCA circulation, such as ischemic stroke and cerebrovascular insufficiency.

## Figures and Tables

**Table 1:** Acupuncture rationale and needling details according to STRICTA guideline.

1. Acupuncture rationale	(i) According to the meridian theory of Traditional Korean medicine
	(ii) Based on historical context, Seven acupoints of stroke
2. Details of needling	(i) 1 needle
	(ii) Dazhui (GV14)
	(iii) 10 mm
	(iv) De-Qi sensation (aching, soreness, pressure, fullness, heaviness, numbness, tingling, warmth, coolness, and dullness sensation)
	(v) Manual stimulation
	(vi) 20 minutes
	(vii) Stainless steel acupuncture (diameter 0.25 mm, length 40 mm, DongBang Acupuncture, Seoul, Korea)
3. Treatment regimen	(i) 2 sessions (once a week)
	(ii) Duration, 1 week
4. Other components of treatment	None
5. Practitioner background	All acupuncture treatments were performed by one experienced Korean medical doctor (Hyun Ku Lee).

**Table 2:** Comparing the CO_2_ reactivity before and after GV14 acupuncture.

	GV14 acupuncture		*p* value^*∗*^
	Before	After	
BA	3.28 (2.18–4.75)	4.70 (3.23–7.19)	<0.001
Right MCA	3.81 (1.83–8.05)	5.25 (2.81–11.58)	0.001
Left MCA	3.84 (2.38–9.21)	5.12 (2.95–8.29)	0.005

Values are median (range). BA, basilar artery; MCA, middle cerebral artery. ^*∗*^*p* values were calculated by Wilcoxon signed-rank test.

**Table 3:** Comparing the CV40 before and after GV14 acupuncture.

	GV14 acupuncture		*p* value^*∗*^
	Before	After	
BA (cm/s)	45.49 (32.70–69.44)	50.41 (33.97–77.19)	0.003
Right MCA (cm/s)	74.08 (39.85–134.34)	78.07 (34.24–173.08)	0.408
Left MCA (cm/s)	68.15 (40.90–120.70)	69.78 (39.63–145.08)	0.234

Values are median (range). BA, basilar artery; MCA, middle cerebral artery; CV40, modified blood flow velocity at *P*_ETCO2_ = 40 mmHg. ^*∗*^*p* values were calculated by Wilcoxon signed-rank test.

**Table 4:** Comparing the mean blood pressure and heart rate before and after GV14 acupuncture.

	GV14 acupuncture		*p* value^*∗*^
	Before	After	
Mean blood pressure (mmHg)	106.83 (94.33–120.33)	107.08 (91.67–118.67)	0.355
Heart rate (bpm)	77 (55–101)	75 (52–94)	0.431

Values are median (range). bpm, beats per minute. ^*∗*^*p* values were calculated by Wilcoxon signed-rank test.

## Data Availability

The data used to support the findings of this study are included within the article.
